# Design and Development of Virtual Medical System Interface Based on VR-AR Hybrid Technology

**DOI:** 10.1155/2020/7108147

**Published:** 2020-08-25

**Authors:** Xu Cong, Tingting Li

**Affiliations:** School of Digital Art and Design, Dalian Neusoft University of Information, Dalian, 116023 Liaoning, China

## Abstract

With the continuous development of information technology and digital medicine, computer-assisted virtual medicine has become the development trend of a new generation of clinical surgery, which aims to improve the accuracy of surgery, reduce the risk of surgery, and achieve precise and minimally invasive treatment. The interface design in the computer-aided virtual medical system is a medium for transmitting and exchanging information between humans and machines. This article uses virtual reality technology and augmented reality technology to develop a virtual medical system interface, which aims to solve the interaction problem between users and virtual medical systems and satisfy users. The multidemand psychology is an effective way of interaction. It provides users with a multichannel and comprehensive communication method, which truly meets the design goals that meet the user's psychological needs. It also expands applications for virtual reality technology and augmented reality technology.

## 1. Introduction

With the increase of China's medical level and the rapid development of mobile Internet, the interactive display method of augmented reality has been favored by more people. Many scholars at home and abroad have carried out related exploration and application research on augmented reality technology. Augmented reality technology can use virtual additional information to enhance the user's observation of the real world, so as to fully stimulate the enthusiasm of the visitors from the perspective of the visitors. However, in actual practice, some problems have been exposed. For example, the immersion is not strong. Users hope that they will have close contact with the models in the scene. A single augmented reality technology weakens the user's observation and perception. In order to overcome these problems, this article integrates virtual reality technology on the basis of augmented reality technology and combines the two. This paper proposes a virtual medical system interface design and development based on AR-VR hybrid technology.

The interface design in the virtual medical system is a medium for transmitting and exchanging information between humans and machines and refers to the overall design of software's human-computer interaction, operation process, and beautiful interface [1]. The friendly interface design not only gives the user a sense of personality and taste but also makes the user's operation process simpler, more comfortable, and free, while fully reflecting the features and functions of the software. In the virtual medical system, users need to implement various operations through its interface, so the interface design is particularly important in virtual medical design [2]. At present, the interface of China's virtual medical system lacks human-system interaction, and the user's multiple needs cannot be met. The interface design of the virtual medical system is relatively single, which is inconvenient for users and increases the user's operating burden [3]. In response to the above problems, in 2005, Pioneer introduced a 3D user interface technology that allowed users to draw text and pictures on a PC without using a keyboard, mouse, or touch screen. This system realizes a completely new feeling atmosphere environment, which is very different from the traditional interface in the past [4]. Enea China Service and R & D Center has launched a three-dimensional stereo operation interface that can be used on smart mobile devices to facilitate users to complete multiple applications more quickly. The interface introduced by Enea mainly includes the main interface, tool interface, movie display interface, and video and audio interface. The picture interface is a five-category interface. This functional interface for different needs can save users' time to use the application and add more entertainment [5].

This article proposes to apply virtual reality technology and augmented reality technology to the interface design of the virtual medical system, presenting a three-dimensional effect to the user, while enjoying different service methods. The discussion of the interface of the virtual medical system is a human-computer interaction method. A new attempt and innovation has theoretical and practical significance for realizing a human-friendly human-machine interface. At the same time, it also expands a wider development space for virtual reality.

## 2. Virtual Reality Technology and Augmented Reality Technology

### 2.1. Virtual Reality Technology

Virtual Reality (VR for short) was formally proposed by Jaron Lanier, founder of the American VPL company, in the early 1980s. As a collection of high technology, virtual reality technology integrates many key technologies, such as multimedia, computer graphics, sensor technology, artificial intelligence, and simulation technology to achieve three-dimensional spatial expression and a natural human-computer interactive operating environment, which has fundamentally changed the status quo of tedious interactions between humans and computers [6]. Virtual reality technology uses a computer to generate a simulation environment with multiple perceptions such as vision, hearing, and touch (such as aircraft cockpits and operation sites), allowing users to invest in the simulation environment through a variety of sensing devices, thereby generating body immediate feelings and experiences [7].

In the research and development of virtual reality technology, most areas are scrambling for research. Virtual reality technology has the advantages of low cost and strong sense of substitution, so it has broad development space. Using glasses connected to smart devices can support the operation of virtual reality experience halls. In the research of virtual reality technology, if it is applied to more complex environments, it will affect the accurate image display of virtual reality technology. Because of this, virtual reality technology needs further research.

Under the virtual reality technology, the glasses equipment must perform focusing, judgment, and other actions and operate according to the user's head. The direction of sound discrimination cannot be effectively applied in virtual reality technology. VR glasses cannot provide users with tactile sensations. Although they have been experienced visually, they cannot actually touch objects, so they are lacking in tactile experience. Information cannot be input in time in the virtual reality technology. The ordinary information input method does not match the virtual reality technology. In this case, the research of VR glasses is extended to data clothing and data gloves. At present, the application range of virtual reality technology is very wide, which has provided a lot of help for aerospace, medical research, and industrial research.

### 2.2. Augmented Reality

Augment Reality (AR) is a new technology developed on the basis of virtual reality technology, which accurately superimposes computer-generated virtual objects or other auxiliary information into the real scene, thereby extending the human sensory experience . The visual perception of the real environment enhances the realism of user experience with the effect of combining reality and reality [8].

The industry first contacted by augmented reality technology is video recording devices, which helps video recording devices collect real-world data and then use virtual scene synthesis technology to synthesize and process them through computers. The application of augmented reality technology is different from the virtual reality technology, and the application is more convenient. You can operate the software by downloading the related software of augmented reality. Of course, this operation experience is not ideal. Augmented reality technology is constantly innovating, combining the principles of optical perspective, integrating the real world with virtual information, and enhancing the content of reality display. The application of this technology requires high investment in cost research, and the technical requirements are very strict.

In recent years, as a cutting-edge technology, augmented reality technology has been widely used in machinery manufacturing, virtual assembly, computer games, multimedia design, simulation training, and other fields [9]. Augmented reality technology continues to innovate, combining the principles of optical perspective, fusion display of the real world and virtual information, and enhanced display content of reality; the application of this technology requires higher investment in cost research; and the technical requirements are very strict. In reality, augmented reality technology mainly includes Google Glass. Of course, because augmented reality technology is in the development and research stage, it needs to be further upgraded in many aspects. Augmented reality technology is involved in military, medical, and industrial fields. Augmented reality technology provides users with accurate information and positioning and assists users to achieve research and operation purposes. At the same time, it has been gradually applied in the field of clinical medicine and has become a research hotspot in this field [10].

### 2.3. Differences between Virtual Reality Technology and Augmented Reality Technology

The visual presentation method of virtual reality technology is to block the connection between the human eye and the real world and render a brand new world in real time through the device, and the virtual reality technology presents all fake and fictional. The visual presentation method of augmented reality technology is to superimpose the holographic image and strengthen the visual presentation method when the human eye is connected with the real world. It is a combination of physical and virtual.

### 2.4. What Can Virtual Reality and Augmented Reality Do

Because virtual reality and augmented reality technology can establish a connection between the virtual world and the real world and form a new mode of human-computer interaction, thereby greatly expanding the human sensory experience and recognizing the world, with the gradual maturity of VR/AR technology, certain applications have been realized, and there is a trend to further form the industry. According to the VR report released by Goldman Sachs, VR and AR not only have the potential to create new markets but will also disrupt some of the current markets. The technology can be applied to 9 major areas: video games, live events, video entertainment, healthcare, real estate, retail, education, engineering, and military.

### 2.5. Opportunities and Challenges of Virtual Reality and Augmented Reality

In the future, VR technology will have greater applications and breakthroughs in the medical field. Specifically, VR technology can be used to generate visual images to check the patient's specific conditions, such as using CAT scans or ultrasound diagnostic images to generate 3D models of patient anatomy. In addition, VR technology can also be used to recover patients. In Europe, immersive virtual display therapy is used in patients with stroke and brain damage in many places to restore their motor and cognitive abilities. Because of the related equipment of VR technology, it still has problems such as inconvenience and poor results, and the processing speed of its hardware is far from meeting the needs of processing large amounts of data in the virtual world in real time. In addition, the price of some equipment is very expensive, and it is difficult to make the equipment universal. Due to the limitations of hardware technology, the development of virtual reality software requires huge expenses, but it cannot achieve the expected application effect. At the same time, there are still many problems in the application of VR technology in high-speed graphics and image processing, artificial intelligence, and other fields, which need to be solved urgently.

At present, AR technology is developing rapidly, and there is much room for development in many fields. AR technology has three main characteristics: integrating real-world and virtual environment information, real-time interactivity, and adding positioning virtual objects in three-digit scale space. However, due to the diversity and complexity of the external environment, AR equipment is often subject to external interference, and there are still great shortcomings in application. Therefore, it is necessary to improve the synthetic tactile input technology of users in the real environment.

## 3. Analysis of the Characteristics of the Interactive Interface Design of the Virtual Medical System

### 3.1. Interactive Interface Concepts

[11]. Interactive interface refers to the medium for communication between people and machines, the medium for exchanging and transmitting information between users and computers, and also a comprehensive operating environment for users to conduct computer operation (i.e. information exchange and communication through this medium)[11]. In the design of interactive interface, it is especially emphasized to be user centered, so it is also called user interface (UI) or human-computer interface.

For individuals, cognition of the real world is actually an electronic signal transmitted from the senses to the brain, and enhanced perception can expand our cognitive world. Just as you can see farther with a telescope and tiny creatures with a microscope, VR/AR technology can present things we could not previously perceive, simulate sounds we cannot hear, and make things unclear clear. And it can create an enhanced version of the real world. From this perspective, VR/AR technology helps us see the world more clearly, more thoroughly, and richer by enhancing our perception.

The invention is aimed at a problem that is not effective in the prior art and can be achieved through human-computer interaction so that patients can truly understand the causes, pathologies, schemes, procedures of surgery, and matters needing attention after surgery, etc., and provides a virtual reality-based interactive medical system.

### 3.2. Basic Elements of Interactive Interface

The basic elements of interactive interface design are divided into several parts, including text, color, graphics, and sound [12]. These basic elements are responsible for providing users with sufficient visual and auditory feelings, and these sensory stimuli are relevant to users. The information is also used as a source of information and auxiliary tools for users during operation. Without these basic elements, users will not be able to understand the meaning and information conveyed by the machine or the interface itself. The central processing unit includes a message receiving and sending module and a VR video storage module for various medical processes and knowledge and patient information identification module; VR glasses and doctors' terminals also set up information receiving and sending modules to achieve connection with the central processor, the doctor's terminal, and the central processing unit of the VR device connected through the information receiving and sending module; and the doctor's terminal selects the appropriate VR video for the patient according to the patient's situation and sends it to the VR device through the information receiving and sending module. The central processor of the VR device retrieves the corresponding VR video from the VR video storage module according to the information sent to the VR glasses, assisted with the screening for patients to learn from human-computer interaction.

### 3.3. Feature Analysis of Interactive Interface of AR Medical System

The basic characteristics of the traditional two-dimensional interactive interface are the interaction between the mouse and the desktop system and design elements based on the Windows operating system. When designing a two-dimensional interactive interface in the desktop era, you only need to consider the window system and two-dimensional visual design elements in the desktop environment. However, when designing an AR-based interactive interface, because its operating environment is a combination of reality and reality, this also determines the complexity of its interactive interface design. Therefore, in specific applications, attention should be paid to how to enable users to effectively interact with AR-based interaction interfaces in complex environmental relationships. Considering the above factors, when designing an AR-based interactive interface, it should pay attention to its three design characteristics: virtual-real superposition, three-dimensionality, and real-time interaction.

#### 3.3.1. Virtual and Real Superposition

Compared with the interactive interface based on augmented reality and the interactive interface based on virtual reality, the interactive environment of the latter is completely virtual. In the former, the user is faced with an information environment in which virtual information and real environment are superimposed. On the other hand, compared with the latter's interactive interface, the former's user experience will be more real and immersive. Among them, the displayed virtual information can be two-dimensional information, such as text and images, and can also be three-dimensional information, such as audio, video, animation, and smell. In the AR-based interactive interface, the real physical environment is superimposed and enhanced by virtual information. Compared with a completely virtual interactive interface or a completely real interactive interface, the AR interactive interface has more room to play in the interactive experience. Sex makes the human-computer interaction experience even more extreme.

#### 3.3.2. Three-Dimensionality

The three-dimensional characteristics are mainly reflected in the three-dimensionality of the interaction mode and the interactive environment. In the AR-based interactive interface, the virtual world and real physical world of the two required elements determine the true three-dimensional properties of the AR interactive interface. What the user feels is a three-dimensional world where the virtual world and the real physical world are superimposed. This immersive three-dimensional environment enables users to obtain a completely new user experience. At the same time, the interaction between the user and the computer also has three-dimensional properties. For example, interaction technologies such as gesture control will form an essential element of the three-dimensional interaction system.

#### 3.3.3. Real-Time Interaction

In the AR interactive interface, users not only are exposed and experienced an interactive interface that mixes virtual information and real environment but also experience the real-time nature of interactive operations. Similar to computer operation, in the operation of AR interactive interface, users essentially obtain and exchange information with computer hardware through the interactive interface, but the difference is that the interaction of augmented reality is the interaction of virtual information and real environment. In order to give the user a realistic user experience, when the user performs interactive operations, it should interact with the augmented reality system in real time. In simple terms, when a user issues an instruction, the system must implement and feedback in time. The response time during this period should be handled as quickly as possible. Therefore, in the AR interactive interface, its real-time interaction is reflected in the two aspects of human-computer interaction and virtual-real interaction. The real environment seen by users through the device must be immediately and in real time superimposed on the virtual information that matches the user's needs.

### 3.4. Analysis of Interactive Interface Features of VR Medical System

Virtual reality technology is a man-machine interface technology that highly realistically simulates human behaviors such as sight, hearing, and touch in the natural environment. American scientist Burdy proposed the “virtual reality technology triangle” theory. The three outstanding features are immersion, interactivity, and imagination.

VR technology is a virtual reality technology, which is currently being applied to human-computer interaction technology. How to effectively apply the technology to the medical system and solve the technical problems of the existing technology, so as to realize the guidance of the medical system through the various procedures of VR technology; to explain the cause of disease, pathology, treatment plan, operation process, postoperative precautions, etc; The existing technologies need to be solved urgently, such as reducing patients' fear of disease, operation and unfamiliar environment in hospital, making operation plan, conducting surgical rehearsal, surgical teaching, surgical skill training, guiding operation during operation and postoperative rehabilitation.

#### 3.4.1. Immersion

. The ideal virtual environment should be to make the user's vision, hearing, touch, smell, and taste extremely realistic, that is, to allow users to immerse themselves in a part of the virtual environment, so that the user is fully placed in the virtual environment. The performance standard of a virtual reality system is the level of immersion. The key to the good and bad of a virtual environment setting is whether to make users feel a very strong immersion.

VR technology allows the real world to be transformed into a virtual world, and AR technology allows the virtual world to be superimposed on the real world, enabling the virtual world and the real world to interact. There is a convergence trend between the two worlds. In the future, you may not be able to tell which part is virtual and which part is real. The whole world becomes a mixture of virtual and real worlds.

#### 3.4.2. Interactivity

Interactivity refers to the degree to which users can manipulate objects and the degree of emotional feedback experience. This interactive mode uses special input and output devices (such as data gloves, data suits and head-mounted displays) to use your own body language to interact naturally with objects in the virtual environment, and the computer can use the user's hands, eyes, head, language and body movements to adjust the virtual image and constantly update the virtual environment to display to the user. For example, users can grab objects directly with their hands in a virtual environment and can feel the weight and at the same time can move with their hands. Therefore, the existing technology needs a system that can realize human-computer interaction and enable patients to experience the real experience to complete the above tasks, in order to save medical resources to the greatest extent and minimize doctor-patient contradictions.

VR/AR technology can reconstruct the information of the physical characteristics of the physical world and build a virtual world as real as the real world. Just as a telephone can convert sound waves into electrical signals and convert them into sound, video phones can reconstruct information from sound and pictures, while “real” virtual worlds reconstruct information from the multidimensional characteristics of objects. If we reconstruct all the information of the physical world and transform it into a virtual space, then your mobile phone can use the Internet of Things technology to fit the entire world, easily put it in your pocket, and see the world without leaving the house.

#### 3.4.3. Conceptual

Imagination, also called creativity, refers to the fact that virtual reality technology has a very wide imaginable space. It not only provides creative ideas for designers to conceive and design virtual environments but also helps humans to obtain more information and knowledge through their own thinking. In short, the three characteristics, immersive, interactive, and imaginative features of the virtual reality system, visually present a virtual space of natural interaction. The virtual environment with these three characteristics allows people to not only feel the immersion of the body but also be spiritually satisfied.

The establishment of multidimensional memories and multidimensional thinking, combined with the assistance of artificial intelligence and the Internet of Things, is likely to trigger a revolution in human learning and break through the physiological limits of the human brain. From word of mouth to text recording to audio and video, each media promotion brings the leaping development of human civilization, and the three-dimensional and interactive learning method produced by VR/AR technology will also promote the progress of human civilization. VR is far more than “vivid” and “lively” in the teaching field, and its significance is more profound in medicine. For example, a beating heart is projected on the screen, and the operator can observe each part at any time and split them. Such teaching not only saves the biological cost of experiments but also improves the accuracy and understanding of students' operations.

## 4. Design and Development of Interactive Interface of Virtual Medical System

### 4.1. Virtual Medical System Development Platform

This system uses Unity 2018.2.16 as the base platform and combines Vuforia to realize the virtual medical system function development. Vuforia is the industry's leading and most widely used augmented reality platform. It can easily and quickly create the interaction between the virtual world and the real world. It uses computer vision technology to identify and track flat images and simple 3D objects in real time. Developers can bridge the real-world and digital experiences.

### 4.2. Virtual Medical System Interface Design

The interface of the virtual medical system is designed in two parts. One uses the Vuforia SDK to implement the function of the augmented reality module of the virtual medical system and scans the picture for tracking. When the picture is scanned by the camera, some set 3D objects appear above the picture. The other part uses the Unity3D platform to build virtual scenes to achieve 3D interactive virtual roaming effects.

### 4.3. Virtual Medical System Interface Development

#### 4.3.1. Augmented Reality Module Development

There are two stages to process target pictures in the development of the augmented reality module of the virtual medical system. First, the target image needs to be designed and then uploaded to the Vuforia platform for target processing and evaluation. In the recognition process, Vuforia compares the natural feature points of the input image with its own feature point database to determine the recognition process. In the Valoria image recognition workflow, you need to use Vuforia Target Manager to generate a feature database of recognition maps.

#### 4.3.2. Virtual Reality Module Development

The virtual reality module in the virtual medical system is developed based on the Unity3D platform. First of all, a virtual medical model needs to be built. The production of this virtual medical model can be done with 3d Max software, and the texture of the model can be designed with Photoshop. This is a very heavy workload. It takes a long time to make the model. Use 3d Max software to make a virtual medical scene, and then put the completed model into Unity3D to synthesize according to the actual proportion. Then, according to the predesigned function, the corresponding code is added to implement the roaming module function of the virtual medical scene.

### 4.4. Virtual Medical System Interface Test

**Figure 1:**
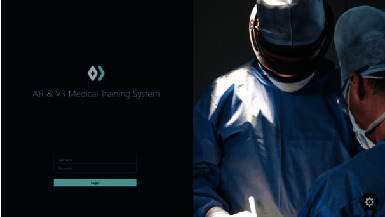
System login screen.

**Figure 2:**
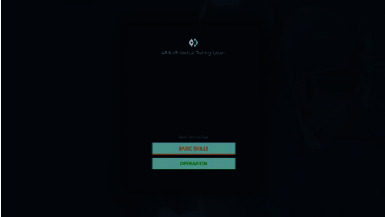
System function selection interface.

**Figure 3:**
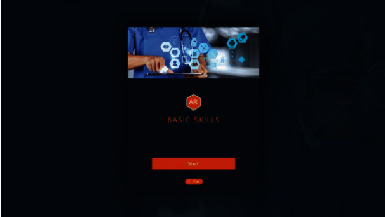
AR system module interface.

**Figure 4:**
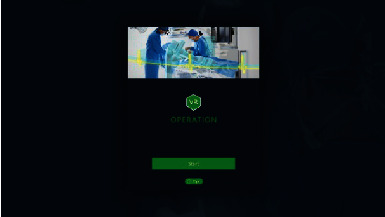
VR system module interface.

The system compilation environment is Visual Studio 2017. The code is written in C # and is based on the Unity3D platform combined with the Vuforia SDK. After starting the system, enter the system login interface, click the instructions to view the system usage method, and enter the account and password to log in to the system, as shown in Figure 1. After entering the system, enter the scene selection mode. There are 2 scenes to choose from, as shown in Figures 2–4. After entering the scene, you can control the third-person position movement in the scene and change the perspective of roaming by touching with your finger, so as to realize individualized autonomous learning of medical knowledge.

### 4.5. VR and AR Technology Utilization in Medical Industry

**Figure 5:**
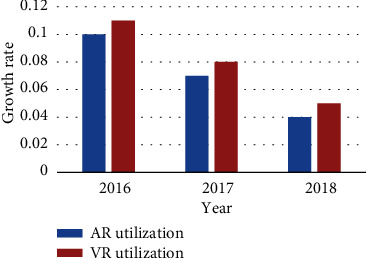
Utilization of VR and AR technologies in the medical community.

At present, as AR and VR technologies are emerging technologies, their application in the medical field is still in an experimental stage. However, once these technologies are applied, they have gained great recognition from the medical community and have provided great impetus to the development of medical treatment. It can be seen from Figure 5 that the utilization rate of VR and AR technology in the medical industry is also increasing. VR technology has increased from 0.05% in 2016 to 0.11 in 2018, more than doubled; AR technology has increased from 0.04% in 2016 to 0.1% in 2018, more than doubled.

### 4.6. Praise Rate of VR and AR Technologies

**Figure 6:**
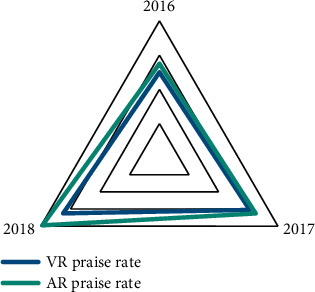
Praise rates for VR and AR technologies.

Due to the rapid development of China's technology in the past two years, the technical levels of AR and VR have also greatly improved, and people's recognition of these two technologies has gradually increased. It can be seen from Figure 6 that AR technology has increased from 55% in 2015 to 79% in 2018; and because AR technology will be affected by environmental factors, the praise rate has grown slowly, from 50% in 2016 to 65% in 2018.

## 5. Conclusions and Outlook

In this paper, by studying the existing design theories and combining the related technology implementation backgrounds of augmented reality and virtual reality technology, this paper discusses and summarizes the theory and design schemes of interactive interface design of augmented and virtual reality for virtual medical systems. Technology and augmented reality technology are applied to home media products, and the user's sense of immersion and experience is improved by constructing virtual scenes, and finally the virtual medical system interactive interface is developed.

At present, the AR/VR industry is still in its infancy, and there are still problems such as insufficient applications, insufficient technical reserves, and insufficient database construction. For example, AR/VR products still have problems in terms of convenience and application popularity, and developers need to make great efforts to find the combination of technology and applications. For AR/VR technology, what ordinary users expect is not a cool technical experience, but a real improvement and improvement in the quality of life and learning level. The current augmented reality technology is still in the development stage, and we have no way to completely rely on augmented reality applications and devices in our lives. For example, VR devices are prone to vertigo. To make matters worse, reliable research has confirmed that the dizziness caused by VR devices may still exist after you stop using the device for 8 hours. Until now, in addition to vision and hearing, virtual reality technology has not been able to simulate the other three senses (olfactory, touch, and taste). Unlike the hardware market that contends, software and content are the shortcomings of AR/VR. VR/AR can really enter the consumer and practical stage. The performance of the GPU needs to be greatly improved in order to meet the needs of our users. For AR/VR technology to be well applied, it is necessary to build a huge virtual reality database and development tools. This is beyond the capabilities of a single enterprise. It requires the state to provide certain funding and policy support to promote the development of technology and the cultivation of the industry.

In the future, with the development of augmented reality technology and the popularization of corresponding display equipment, its display method and effect will certainly have great progress and development than now, the effect of design and production will be more realistic, more in line with the habits of users, so that people can get more diversified and rich information.

## Data Availability

No data were used to support this study.
